# Down‐regulation of miR‐200c attenuates AngII‐induced cardiac hypertrophy via targeting the MLCK‐mediated pathway

**DOI:** 10.1111/jcmm.14135

**Published:** 2019-01-25

**Authors:** Shan Hu, Mian Cheng, Xin Guo, Shun Wang, Beilei Liu, Hong Jiang, Congxin Huang, Gang Wu

**Affiliations:** ^1^ Department of Cardiology Renmin Hospital of Wuhan University, Cardiovascular Research Institute, Wuhan University, Hubei Key Laboratory of Cardiology Wuhan China; ^2^ Department of Geriatrics Tongji Hospital, Tongji Medical College, Huazhong University of Science and Technology Wuhan China; ^3^ Department of Cardiology The Central Hospital of Wuhan, Tongji Medical College, Huazhong University of Science and Technology Wuhan China

**Keywords:** apoptosis, cardiac hypertrophy, miR‐200c, myosin light chain kinase, reactive oxygen species

## Abstract

**Background:**

MicroRNAs (miRNAs) have been shown to commonly contribute to cardiac hypertrophy (CH). The aim of this study was to test the hypothesis that miR‐200c plays an important role in the progression of CH by targeting myosin light chain kinase (MLCK/MYLK).

**Methods and results:**

Cardiac hypertrophy was induced by aortic banding (AB) in rats. Cellular hypertrophy in neonatal rat cardiomyocytes (NCMs) was induced by AngII treatment. Echocardiography, histology and molecular measurements were used to assess the results of the experiments. The levels of apoptosis and reactive oxygen species (ROS) were also measured. Quantitative real‐time PCR (qRT‐PCR) and Western blotting were used to measure mRNA and protein levels respectively. The present results showed that miR‐200c expression was increased in response to CH both in vivo and in vitro. The down‐regulation of miRNA‐200c by a specific inhibitor markedly ameliorated CH resulting from AngII treatment, and the mRNA levels of atrial natriuretic peptide, brain natriuretic peptide and β‐myosin heavy chain were simultaneously decreased. Notably, minimal apoptosis and ROS accumulation were identified in AngII‐induced hypertrophic cardiomyocytes. Conversely, the up‐regulation of miR‐200c using specific mimics reversed these effects. Mechanistic investigations demonstrated that the MLCK gene is a direct target of miR‐200c; an increase in miR‐200c levels led to a decrease in the expression of MLCK and its downstream effector, p‐MLC2, while miR‐200c inhibition increased the expression of these proteins. Furthermore, inhibiting MLCK impaired the anti‐hypertrophic effects contributions produced by the knockdown of miR‐200c.

**Conclusion:**

Our studies suggest that miR‐200c may serve as a potential therapeutic target that could delay hypertrophy. We have also uncovered a relationship between miR‐200c and MLCK, identifying MLCK as a direct mediator of miR‐200c.

## 1 INTRODUCTION

Cardiac hypertrophy (CH) can be defined as either pathological or physiological hypertrophy. Physiological CH, which responds to stimuli such as exercise, is considered adaptive and beneficial. Conversely, pathological CH is caused by pathological stimuli such as high blood pressure, neurohumoral overactivation or other myocardial injury and is maladaptive.^1^ Pathological CH is usually related to complicated pathological processes including oxidative stress, cell apoptosis, inflammation, metabolic dysfunction, sarcomere disorganization and endoplasmic reticulum stress.^2^ Extensive studies have suggested that oxidative stress, inflammation and apoptosis play crucial regulatory roles in CH.^3^, ^4^, ^5^ Additionally, the excessive production of reactive oxygen species (ROS) has been found to result in cardiac dysfunction or injury.^6^ In addition, the inhibition of inflammation and apoptosis has been shown to improve cardiac function.^7^ However, sustained or excessive hypertrophic responses may lead to a transition from compensated hypertrophy to decompensation and, eventually, heart failure (HF), which is associated with sudden death.^8^

Myosin light chain kinase (MLCK), also known as the MYLK, is involved in the pathology of several cardiovascular disorders such as HF,^9^ myocardial infarction (MI)^10^ and CH.^11^, ^12^ Cardiac MLCK (cMLCK), encoded by the *MYLK3* gene, is a Ca^2+^/calmodulin‐activated, serine/threonine‐specific protein kinase that phosphorylates cardiac myosin regulatory light chain (cMLC2), which potentiates the rate and the force of contraction in cardiac myocytes.^13^, ^14^ Previously, studies have shown that the increased MLC2 phosphorylation by itself does not cause CH and, in actuality, likely inhibits CH by contributing to enhanced contractile performance and efficiency.^15^

MicroRNAs (miRNAs, miRs) belong to a class of endogenous small non‐coding RNAs (an average size of 22 nucleotides) that negatively regulate the expression of target genes through binding to the 3′ untranslated region within miRNA targets.^16^ MicroRNAs are critically involved in heart function and heart dysfunction in a number of physiological and pathophysiological conditions such as MI, cardiac arrhythmia, CH and HF.^17^ A recent study reported that MLCK in breast cancer cell lines is regulated by miR‐200c, which suppresses epithelial mesenchymal transition during cancer invasion and metastasis.^18^ Moreover, miR‐200c is abundantly expressed in the heart and, in the diabetic heart, is involved in myocardial injury induced by glucose fluctuations that, result in an increase in the levels of ROS.^19^ On the basis of these findings, we suggested a possible regulatory role for miR‐200c in MLCK expression and in the underlying mechanisms of CH.

## 2 MATERIALS AND METHODS

### 2.1 Animals model of aortic banding

All animal care and experiments were performed according to the Guidelines for the Care and Use of Laboratory Animals published by the United States National Institutes of Health (NIH Publication, revised 2011) and the institutional guidelines of the Animal Care and Use Committee of Renmin Hospital of Wuhan University (Wuhan, China). Six‐week‐old adult male Sprague‐Dawley rats weighing approximately 200‐250 g were purchased from the Experimental Animal Center of Wuhan University. The pressure‐overload CH was induced in the rats by transverse abdominal aortic constriction as described previously.^20^, ^21^ In brief, all animals were anaesthetized with chloral hydrate (300 mg/kg, ip). Aortic banding was created around the abdominal aorta using a 7‐0 silk suture and a 22‐gauge needle. The needle was removed, yielding an outer aortic diameter of approximately 0.3 mm. Sham‐operated rats underwent the same procedure but without aortic constriction. At 4 weeks after surgery, cardiac function was evaluated by echocardiography, and samples of the heart tissue were obtained.

### 2.2 Echocardiography

Four weeks after the aortic banding (AB) operation, the rats were anaesthetized with 1.5%‐2% isoflurane via inhalation. Transthoracic echocardiography was performed with an echocardiography machine (iE33, Philips) equipped with a 15‐MHz transducer in order to evaluate CH in rats. Two‐dimensional, guided M‐mode tracings were recorded from the parasternal short‐axis view at the mid‐papillary muscle level.^22^ Interventricular septal end‐diastolic thickness (IVSd), left ventricular posterior wall end‐diastolic thickness (LVPWd) and left ventricular end‐diastolic volume (LVEDV) were measured using three parasternal long‐axis views. Left ventricular fractional shortening (FS) and ejection fraction (EF) were calculated determined by the system and used as direct indicators of cardiac function. All parameters were collected from at least three heartbeats measurements and averaged.

### 2.3 Histological analysis

After echocardiography detection, the rats were killed by cervical dislocation according to the Guide for the Care and Use of Laboratory Animals published by the United States National Institutes of Health. Then, the rat hearts were perfused with phosphate‐buffered saline (PBS) followed by 4% paraformaldehyde (PFA) for global morphometry. For histological analysis, the heart tissues were fixed in 10% formalin, embedded in paraffin or frozen in liquid nitrogen, sectioned at 5‐µm thickness and then stained with haematoxylin and eosin (HE). To evaluate CH, a random collection of 10 cardiomyocytes images which contained at least 20 cells from the cardiomyocyte cross‐sectional area (CSA) was calculated using a quantitative digital image analysis system (Image‐Pro Plus 6.0).

### 2.4 Neonatal rat cardiomyocyte culture

Neonatal rat cardiomyocytes (NCMs) were isolated and cultured from the ventricles of 0‐ to 3‐day‐old Sprague‐Dawley rats as previously described.^22^ In detail, all neonatal rats were killed by decapitation, and their hearts were quickly removed, homogenized and placed in a dish with ice‐cold PBS. After these procedures, NCMs were digested with 0.08% collagenase type II (Sigma, USA) and 0.125% trypsin (Gibco, USA) at 37°C. Then, the cells were centrifuged, followed by differential preplating to enrich the cardiomyocyte population. Next, the cells were cultured in DMEM/F‐12 (Gibco) containing 10% foetal bovine serum (FBS) (Gibco), 0.1 mmol/L bromodeoxyuridine (Sigma) and 1% penicillin/streptomycin (HyClone, USA) in a humidified incubator at 37°C with 5% CO_2_ and 95% air.

### 2.5 Treatments and transfection

Commercially synthesized miRNA‐200c mimics, inhibitor and scrambled control (Table 1) were purchased from GenePharma (Shanghai, China) and transfected into cardiomyocytes using Lipofectamine^TM^ 2000 (Invitrogen) according to the manufacturer's protocol.^23^ Briefly, cells were plated into 6‐well plates with serum‐free DMEM and starved for 12 hours; then, the miRNA‐200c mimics (50 nmol/L) or inhibitor (100 nmol/L) and 5 μL of transfection reagent were separately diluted in 250 μL of OPTI‐MEM (Gibco) and incubated for 5 minutes. Next, the Lipofectamine‐miRNA was incubated for 20 minutes at room temperature and was added to the serum‐free medium. After 6 hours of transfection, the culture medium was replaced with fresh medium containing 10% FBS. The cells were treated with 1 μmol/L AngII or 10 μmol/L ML‐7 (a specific inhibitor of MLCK; Sigma) for 48 hours and then collected for further analysis.

**Table 1:** List of miRNA‐200c sequences

miRNA (GenePharma)	Sequence (5′‐3′)
rno‐mir‐200c inhibitor	CAUCAUUACCCGGCAGUAUUA
inhibitor negative control	CAGUACUUUUGUGUAGUACAA
rno‐mir‐200c mimics	sense: UAAUACUGCCGGGUAAUGAUG anti‐sense: UCAUUACCCGGCAGUAUUAUU
mimics negative control	sense: UUCUCCGAACGUGUCACGUTT anti‐sense: ACGUGACACGUUCGGAGAATT

### 2.6 Immunofluorescent staining for α‐actinin and cell surface area assay

The surface area of the cardiomyocytes was determined using immunofluorescent staining for α‐actinin.^22^ After treatment for 48 hours, the cells were fixed in cold 4% PFA for 20 minutes, permeabilized with 0.5% Triton X‐100 in PBS solution for 10 minutes and blocked in 10% goat serum in PBS for 1 hour at room temperature. Then, the cardiomyocytes were incubated with anti‐actinin primary antibody (at 1:500; Sigma) at 4°C overnight. The cells were then washed with PBS three times and incubated with Alexa Fluor‐555 (Molecular Probes, Eugene, OR) secondary antibody for 1 hour. Subsequently, 4′,6‐diamidino‐2‐phenylindole (1:1000; Sigma‐Aldrich, USA) was used to stain the cell nuclei. Finally, cell images were obtained using a fluorescence microscope (Olympus, Tokyo, Japan), and the CSA was analysed using software Image‐Pro Plus 6.0.

### 2.7 Evaluation of apoptosis

The percentage of cells undergoing early apoptosis and necrosis was measured using a FITC‐Annexin V/PI apoptosis kit (Beyotime, Nanjing, China) using flow cytometry according to the manufacturer's instructions.^24^ After incubation, the cells were washed twice with cold PBS, harvested and were incubated with 5 µL of FITC‐Annexin V and 1 µL of PI working solution (100 µg/mL) for 15 minutes in the dark at room temperature. Cellular fluorescence was measured using a flow cytometer (FACSCalibur, BD Biosciences, CA, USA).

### 2.8 Detection of intracellular ROS production

Intracellular ROS production was determined using a 2,7‐dichlorodihydrofluorescein diacetate (DCFH‐DA) kit (Beyotime) according to the manufacturer's protocol.^25^ The cells were washed twice with PBS, incubated in serum‐free medium containing 10 μmol/L DCFH‐DA for 30 minutes at 37°C and washed twice with PBS. Then, images of the cells were captured using a fluorescence microscope. Finally, the cells were collected for flow cytometry (BD Biosciences, San Jose) to measure the percentage of DCFH‐DA‐positive cells. Following treatment, the malondialdehyde (MDA) concentration and superoxide dismutase (SOD) activity of NCMs in each group were determined using the appropriate kits (Beyotime) according to the manufacturer's instructions.

### 2.9 Luciferase reporter assay

To confirm that MLCK was targeted by miR‐200c, the full‐length 3′‐UTR of rat MLCK/MYLK containing either the predicted wild‐type (WT) target binding site (pmir‐MYLK‐3′‐UTR) or a mutant version (MYLK 3′‐UTR‐MT) was cloned into the pmirGLO luciferase vector (Promega, USA). HEK 293 cells were cotransfected with either the wild‐type or mutant MLCK 3′‐UTR and the pRL‐SV40 Renilla luciferase control vector in the presence of either the miR‐200c mimics (50 nmol/L) or the miRNA control using Lipofectamine 2000 (Invitrogen, NY, USA) for 48 hours. The luciferase activity was assayed using a dual‐luciferase reporter assay system (Promega) and was expressed as a percentage of the luciferase activity of the vector control group.

### 2.10 Quantitative real‐time PCR

Total RNA was extracted from cardiomyocytes using TRIzol (Invitrogen) as previously described. cDNA was synthesized using the Transcriptor First Strand cDNA Synthesis Kit (Roche). The mRNA levels of atrial natriuretic peptide (ANP), brain natriuretic peptide (BNP) and β‐myosin heavy chain (β‐MHC) were quantified using SYBR Green PCR Master Mix (Roche) on an ABI‐7900 Real‐Time PCR Detection System. U6 and glyceraldehyde‐3‐phosphate dehydrogenase (GAPDH) levels were used as references for miR‐200c and mRNA expression. Quantitative real‐time PCR (qRT‐PCR) was performed with the SYBR Green PCR Master Mix (Roche) to determine the levels of our genes of interest, calculated using the comparative quantification method (the expression levels of mRNA = 2^–ΔΔCT^). ΔCT = CT(target gene) − CT(GAPDH/U6), ΔΔCT = ΔCT(experimental group) − ΔCT(control group). The following primers sequences were used:

ANP forward 5′‐GGGAAGTCAACCCGTCTCAG‐3′ and reverse 5′‐CTTCGGTACCGGAAGCTGTT‐3′； BNP forward 5′‐TTCTGCTCCTGCTTTTCCTT‐3′ and reverse 5′‐GCCATTTCCTCTGACTTTTC‐3′； β‐MHC forward 5′‐GATGGTGACACGCATCAACG‐3′ and reverse: 5′‐CCATGCCGAAGTCAATAAACG‐3′； miRNA‐200c forward: 5′‐TGCGCTAATACTGCCGGGTAA‐3′ and reverse: 5′‐CCAGTGCAGGGTCCGAGGTATT‐3′ GAPDH forward: 5′‐CGCTAACATCAAATGGGGTG‐3′ and reverse: 5′‐TTGCTGACAATCTTGAGGGAG‐3′; and U6 forward 5′‐CGCTTCGGCAGCACATATAC‐3′ and reverse 5′‐AAATATGGAACGCTTCACGA‐3′.

### 2.11 Western blot analysis

Cardiomyocytes were collected and lysed in RIPA lysis buffer (Beyotime). The protein concentration was determined using a BCA Protein Assay kit (Beyotime). All of the proteins were separated by 10% SDS‐polyacrylamide gels (Invitrogen) and transferred to polyvinylidene fluoride membranes (Millipore, Billerica, MA, USA), followed by blocking in 5% skim milk in TBST (tris, buffer, solution, tween) for 1 hour at room temperature. Subsequently, the membranes were incubated overnight at 4°C with the following primary antibodies: MLCK (1:1000 dilution, bs‐9865R; Bioss, China), MLC2 (1:2000 dilution, F109.3E1; Enzo Life Science, Farmingdale, USA), p‐MLC2 (1:1000 dilution, #3671; Cell Signaling Technology, MA, USA), cleaved caspase‐3 (1:500 dilutions, NB100‐56113; Novus Biologicals, USA), Bax (1:2000 dilution, #2772, Cell Signaling Technology), Bcl‐2 (1:2000 dilution, ab59348; Abcam, MA, USA) and GAPDH (1:10000 dilution, ab37168; Abcam). Next, the membranes were incubated with the appropriate horseradish peroxidase‐conjugated secondary antibody for 1 hour at 37°C. The proteins were scanned and detected using a FluorChem E System (Cell Biosciences), and ImageJ software was used to determine the intensity of each protein band.

### 2.12 Statistical analysis

All data are presented as the means ± SD of at least three repeated individual experiments for each group. An unpaired, two‐tailed Student's *t* test or one‐way anova, followed by Tukey's post hoc test, was used for the statistical comparisons of two or more than two groups respectively. A *P*‐value <0.05 was considered to denote statistical significance. The data were analysed using spss version 22.0 software (SPSS, Chicago, IL).

## 3 RESULTS

### 3.1 miR‐200c was up‐regulated in the heart tissue of AB rats and in AngII‐induced hypertrophic cardiomyocytes

**Figure 1:**
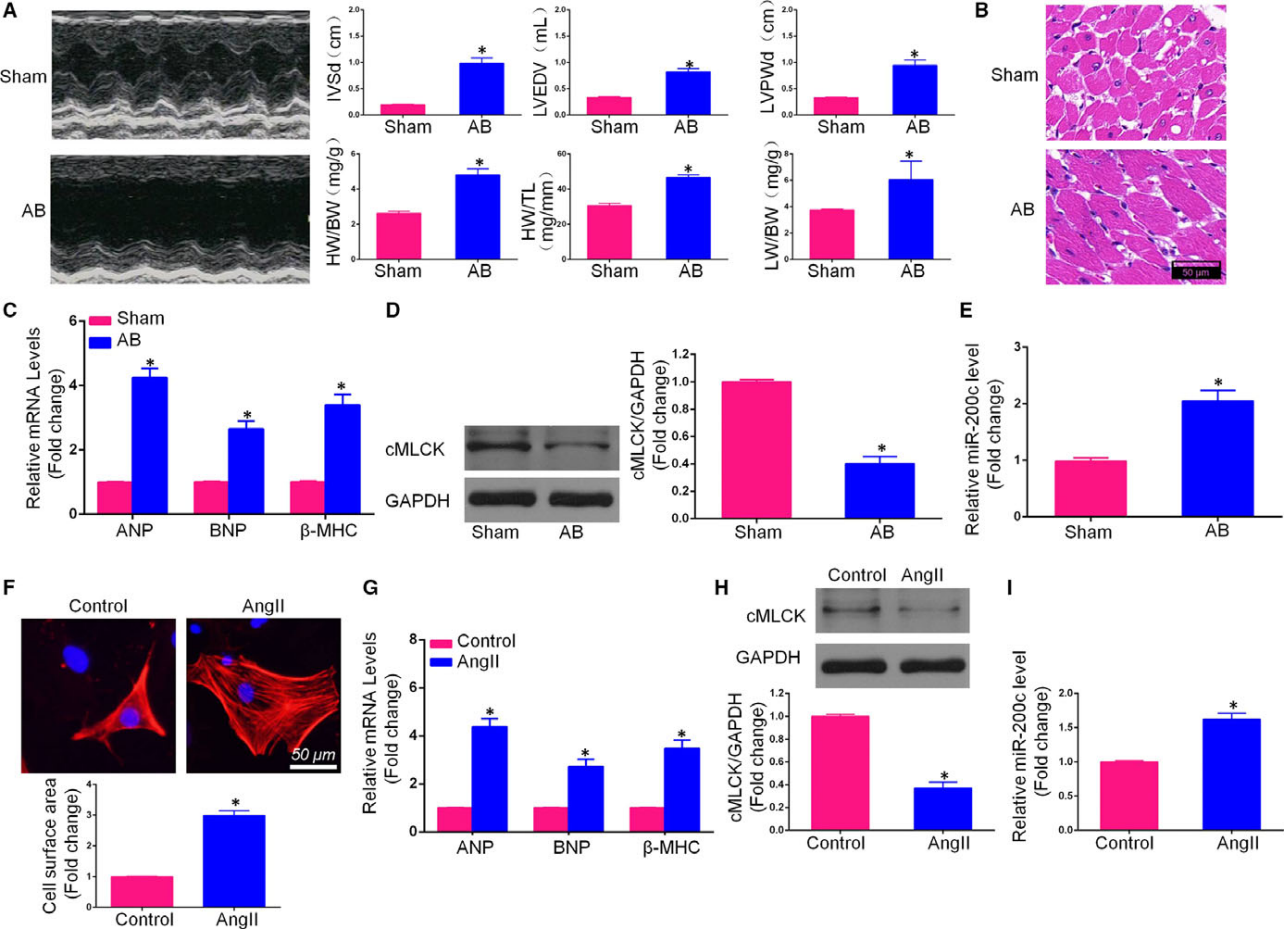
miR‐200c was up‐regulated in the heart tissue of aortic banding (AB) rats and AngII‐induced hypertrophic cardiomyocytes. A, Cardiac hypertrophy indicators, as determined by echocardiography (IVSd, LVEDV and interventricular septal end‐diastolic thickness [LVPWd]) and gross morphology (heart weight/body weight [HW/BW], heart weight/tibial length [HW/TL] and lung weight/body weight [LW/BW]) in the Sham and AB rats; B, Histological analysis of hearts using haematoxylin and eosin staining (scale bar = 50 μm); C, mRNA expression of the hypertrophic markers atrial natriuretic peptide (ANP), brain natriuretic peptide (BNP) and β‐myosin heavy chain (β‐MHC) in the heart tissue of Sham and AB rats; D, Western blot of cardiac myosin light chain kinase (cMLCK) expression in Sham and AB rats; E, the miR‐200c level in the myocardium was up‐regulated in AB rats; F, An immunofluorescence assay for α‐actinin was performed to identify cardiomyocytes (scale bar = 50 μm) and the cell surface area of neonatal rat cardiomyocytes (NCMs) treated with AngII (n = 100+ cells); G, ANP, BNP and β‐MHC mRNA expression, as measured by qRT‐PCR; H, cMLCK expression in hypertrophic NCMs, as determined by Western blot assay; I, The expression level of miR‐200c in cardiomyocytes with or without AngII treatment. (All data are displayed as the means ± SD; (A‐E) AB group, n = 6; Sham group, n = 6; *P < 0.05 vs Sham; (F‐I) n = 4, *P < 0.05 vs Control)

To investigate whether miR‐200c is involved in the progression of CH, we established an AB rat model and a model of AngII‐induced primary cardiomyocyte hypertrophy. Four weeks after the AB surgery, the AB group exhibited increased heart weight/body weight (HW/BW), lung weight/body weight (LW/BW) and heart weight/tibial length (HW/TL) ratios compared with the sham group (Figure 1A). Additionally, transthoracic echocardiography revealed that IVSd, IVSs, LVPWd and LVEDV were markedly increased in the AB group compared with the sham group, and FS% and EF% were significantly decreased in the AB group. Meanwhile, HE staining revealed that the CSA was clearly increased following the AB operation in the AB group compared with the sham group. The gene markers of CH, including ANP, BNP and β‐MHC, were measured in the hearts of the AB rats, which confirmed that the AB operation was successful in inducing CH in rats. Moreover, we observed that cMLCK protein expression was decreased in the AB rat heart tissue compared with that in the sham group (Figure 1). Simultaneously, miR‐200c was up‐regulated in the AB group in comparison with the sham group.

Additionally, NCMs were treated with AngII or PBS and cultured in vitro for 48 hours to induce a hypertrophic phenotype; compared with the control group, the surface area of the NCMs treated with AngII was increased. The mRNA expression levels of ANP, BNP and β‐MHC were also substantially up‐regulated in the AngII‐treated group compared with the control group. Similarly, the protein level of cMLCK was decreased, and the mRNA level of miR‐200c was increased in AngII‐induced hypertrophic cardiomyocytes.

### 3.2 Down‐regulation of miR‐200c attenuated hypertrophy in cardiomyocytes induced by AngII

**Figure 2:**
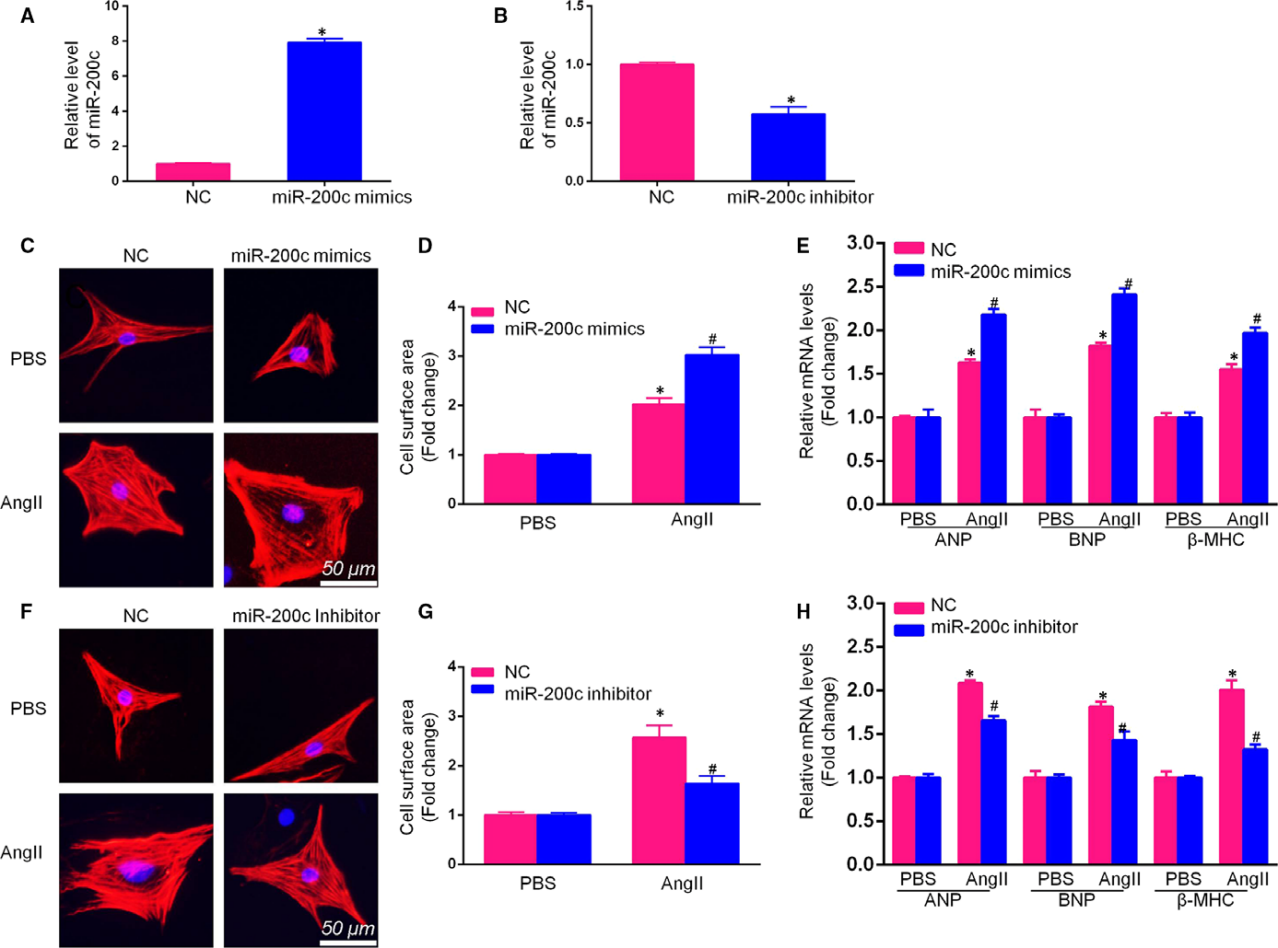
Down‐regulation of miR‐200c attenuated AngII‐induced cardiomyocyte hypertrophy. A, The miRNA level of miR‐200c in neonatal rat cardiomyocytes (NCMs) after transfection with miR‐200c‐specific mimics (50 nmol/L) for 48 h, as detected by qRT‐PCR; B, The miRNA level of miR‐200c in NCMs after transfection with miR‐200c‐specific inhibitor (100 nmol/L) for 48 h, as detected by qRT‐PCR; (C,D) Representative images and cross‐sectional area (CSA) of NCMs transfected with miR‐200c mimics or miR‐200c negative control (NC) and treated with AngII for 48 h (scale bar = 50 μm); E, The relative mRNA levels of atrial natriuretic peptide (ANP) and β‐myosin heavy chain (β‐MHC) in NCMs transfected with miR‐200c mimics or NC mimics and treated with AngII or phosphate‐buffered saline (PBS) for 48 h; F, Representative images and CSA of NCMs transfected with miR‐200c inhibitor or miR‐200c NC and treated with AngII or PBS for 48 h (scale bar = 50 μm) (n = 100+ cells); G, The relative mRNA levels of ANP and β‐MHC in NCMs transfected with miR‐200c inhibitor or NC inhibitor and treated with AngII or PBS for 48 h. (All data are displayed as the means ± SD; n = 4 samples per experimental group; *P < 0.05 vs NC mimics or NC inhibitor/PBS; ^#^ *P* < 0.05 vs NC mimics or NC inhibitor/AngII)

To assess whether miR‐200c regulates CH, miR‐200c specific mimics or inhibitors were transfected into AngII‐stimulated cardiomyocytes for 48 h. The qRT‐PCR results revealed that the cells transfected with miR‐200c mimics (50 nM) presented with an up‐regulated expression of miR‐200c, whereas the cells transfected with the miR‐200c inhibitor (100 nM) had reduced miR‐200c levels (Figure 2A).

The quantification of the ANP, BNP and β‐MHC mRNA levels and NCM surface area assays were used to evaluate the hypertrophic cardiomyocytes. Compared to miR‐NC (negative control)‐treated cells, the overexpression of miR‐200c in cardiomyocytes significantly increased, lead to a significant increase in the mRNA levels of ANP, BNP and β‐MHC and increased the cell surface area. In contrast, the down‐regulation of miR‐200c by the inhibitor substantially reduced the mRNA levels of ANP, BNP and β‐MHC (Figure 2D) and decreased the surface area of NCMs after AngII treatment. These results suggest that the down‐regulation of miR‐200c attenuated CH.

### 3.3 miR‐200c inhibition suppressed CH via the regulation of apoptosis

**Figure 3:**
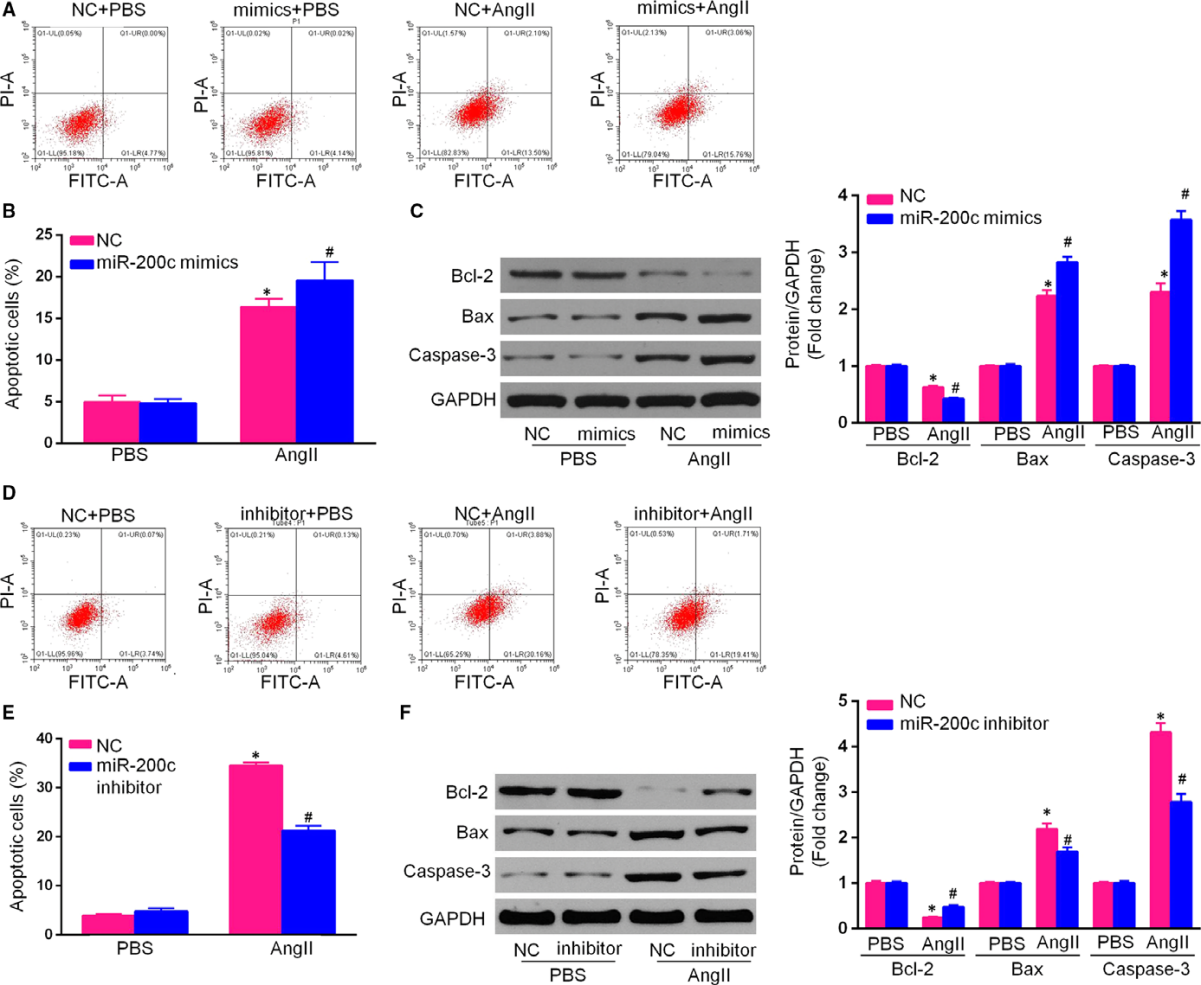
MiR‐200c inhibition promoted cardiac hypertrophy via regulation of apoptosis. (A,B) Cell apoptosis was detected by flow cytometric analysis, and the percentage of apoptotic cells transfected with miR‐200c mimics or NC mimics and treated with AngII or phosphate‐buffered saline (PBS) for 48 h was evaluated; (C,D) The protein expression levels of Bcl‐2, Bax and cleaved caspase‐3 were detected in the different groups using Western blotting and were normalized to the housekeeping gene GAPDH; (E,F) Cell apoptosis was detected by flow cytometric analysis, and the percentage of apoptotic cells transfected with miR‐200c inhibitor or NC inhibitor and treated with AngII or PBS for 48 h was determined; (G,H) The protein expression levels of Bcl‐2, Bax and cleaved caspase‐3 were detected in the different groups using Western blotting and were normalized to the housekeeping gene GAPDH. (All data are presented as the means ± SD; n = 4 samples per experimental group; *P < 0.05 vs NC mimics or NC inhibitor/PBS; ^#^ *P* < 0.05 vs NC mimics or NC inhibitor/AngII)

We examined the apoptotic effects of miR‐200c in hypertrophic cardiomyocytes by measuring the percentage of early apoptotic and necrotic cells using a FITC‐Annexin V/PI apoptosis assay. As shown in Figure 3A and B, the apoptotic percentage was increased in NCMs treated with AngII compared to those treated with PBS. Additionally, compared to transfection with miR‐NC, transfection with miR‐200c mimics successfully increased the apoptotic percentage after exposure to AngII for 48 hours. In contrast, compared with miR‐NC transfection, transfection with the miR‐200c inhibitor significantly reduced the percentage of apoptotic NCMs. Western blotting analysis was used to evaluate the expression levels of the pro‐apoptotic makers Bax and cleaved caspase‐3 and of the anti‐apoptotic marker Bcl‐2 in AngII‐induced cardiomyocytes. Figure 3C shows that miR‐200c up‐regulation increased the levels of Bax and cleaved caspase‐3 and decreased the level of Bcl‐2. However, cardiomyocytes transfected with miR‐200c inhibitor had notably reduced levels of Bax and cleaved caspase‐3, while the level of Bcl‐2 was markedly decreased. As demonstrated above, the miR‐200c inhibitor strongly inhibited apoptosis in AngII‐induced cardiomyocytes.

### 3.4 miR‐200c modulated CH via regulation of ROS

**Figure 4:**
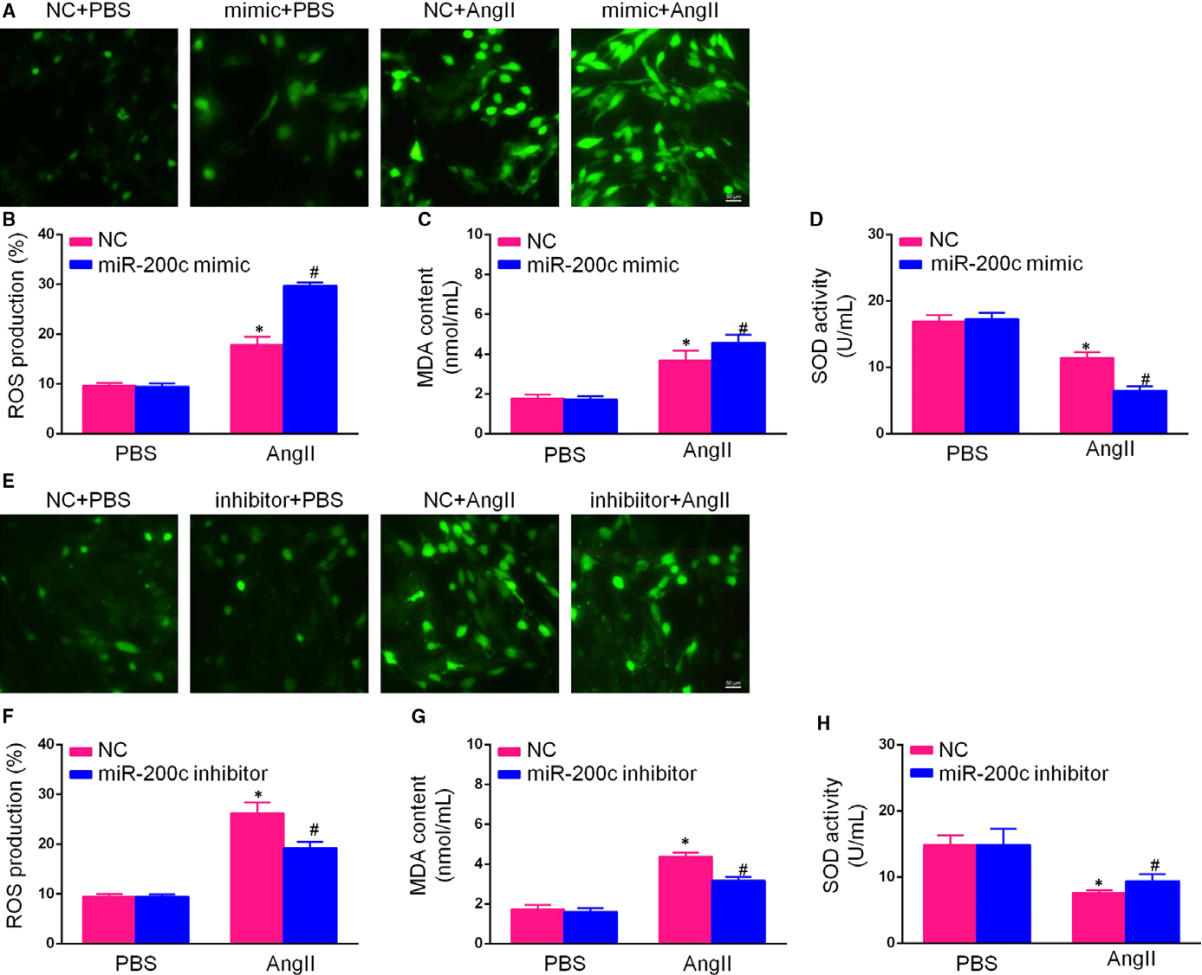
MiR‐200c‐mediated cardiac hypertrophy was associated with reactive oxygen species (ROS) regulation. (A,B) Representative photomicrographs showing ROS accumulation, as determined using the 2,7‐dichlorodihydrofluorescein diacetate (DCFH‐DA) probe in neonatal rat cardiomyocytes (NCMs) transfected with a miR‐200c mimics or NC mimics and treated with AngII or phosphate‐buffered saline (PBS) for 48 h (scale bar = 50 μm); (C,D) Relative levels of malondialdehyde (MDA) content and superoxide dismutase (SOD) activity in NCMs transfected with miR‐200c mimics or NC mimics and treated with AngII or PBS for 48 h; (E,F) Representative photomicrographs showing ROS accumulation, as determined by the DCFH‐DA probe under a fluorescence microscope in NCMs transfected with miR‐200c inhibitor or NC inhibitor and treated with AngII or PBS for 48 h (scale bar = 50 μm); (G,H) Relative levels of MDA content and SOD activity in NCMs transfected with miR‐200c inhibitor or NC inhibitor and treated with AngII or PBS for 48 h. (All data are displayed as the means ± SD; n = 4 samples per experimental group; *P < 0.05 vs NC mimics or NC inhibitor/PBS; ^#^ *P* < 0.05 vs NC mimics or NC inhibitor/AngII)

We next assessed whether miR‐200c is involved in the regulation of reactive oxidative stress in AngII‐induced hypertrophic cardiomyocytes using DCFH‐DA (for H_2_O_2_^−^) probes to measure ROS production in cardiomyocytes transfected with miR‐200c mimics/inhibitor or NCMs after treatment with AngII or PBS for 48 hours. As shown in Figure 4, the overexpression of miR‐200c significantly increased ROS production, while the down‐regulation of miR‐200c expression decreased ROS production in AngII‐induced cardiomyocytes. Furthermore, flow cytometric analysis was used to determine the mean fluorescence intensity values. Compared to transfection with miR‐NC, transfection of miR‐200c mimics successfully increased ROS production induced by 48 hours of AngII treatment for, while transfection with the miR‐200c inhibitor transfection abolished this change in ROS production.

In addition to ROS production, the oxidative stress markers MDA and SOD were also investigated. The production of MDA, which is an end product of lipid peroxidation, was used to measure the extent of oxidative stress. The activity of SOD, which is an antioxidant enzyme, was decreased during CH. The MDA was substantially increased in the miR‐200c mimic group compared to the miR‐NC group after AngII exposure, which is consistent with a reduction in SOD activity. Moreover, the miR‐200c inhibitor reversed these effects in hypertrophic cardiomyocytes, as demonstrated by a reduction in MDA level and an increase in SOD activity. Overall, these results suggest that, miR‐200c plays a key role in the regulation of ROS during the development of CH.

### 3.5 miR‐200c directly targeted MLCK

**Figure 5:**
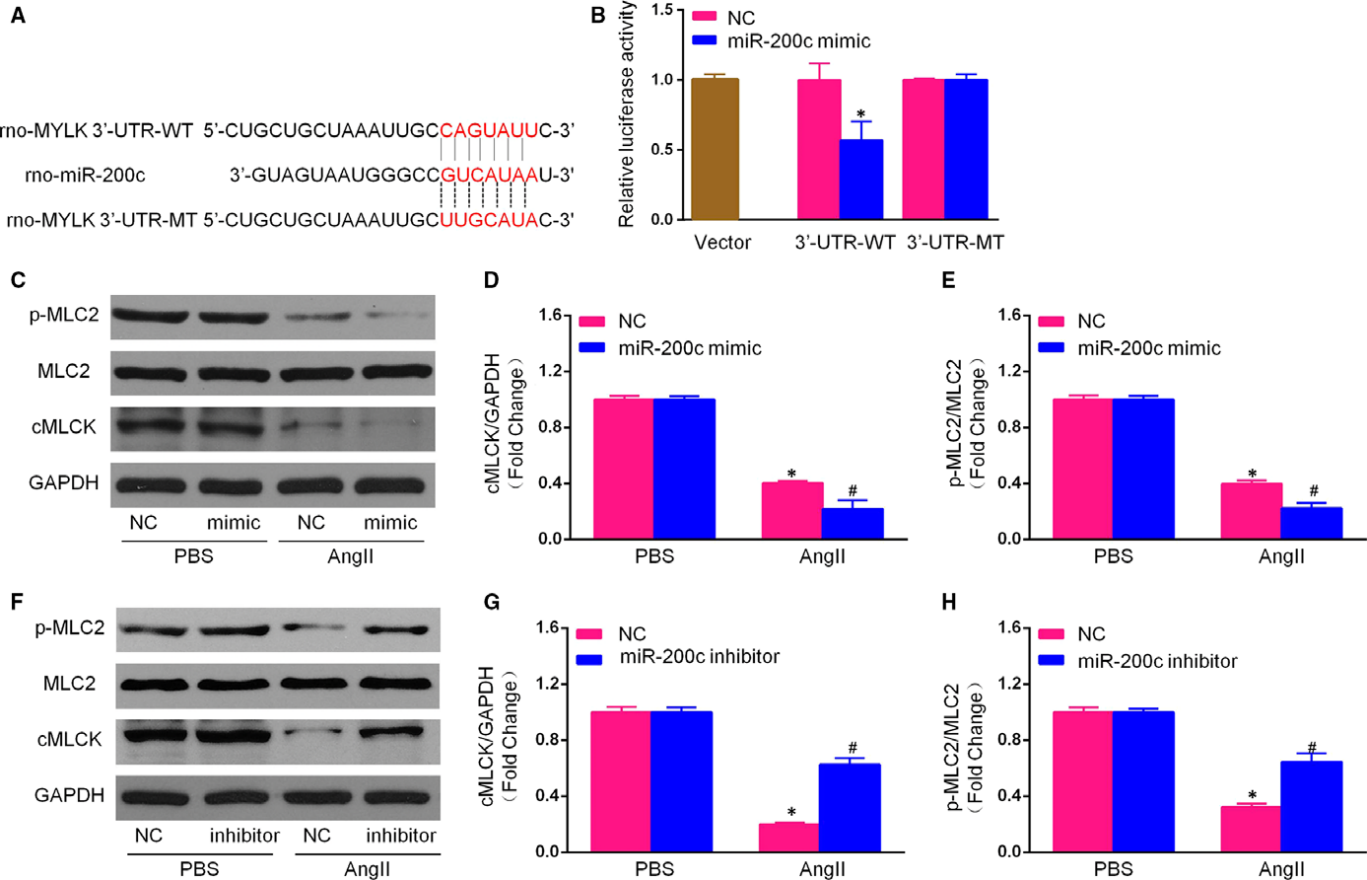
MiR‐200c directly targeted myosin light chain kinase (MYLK/MLCK). A, The sequence of rat miR‐200c was aligned with the WT and mutant 3′‐UTR of MYLK/MLCK; B, Luciferase assays revealed that miR‐200c targeted the 3′‐UTR of MYLK in HEK 293 cells 48 h after transfection; (C‐E) The protein expression levels of cardiac myosin light chain kinase (cMLCK), myosin regulatory light chain (MLC2) and p‐MLC2 were detected using Western blotting in NCMs transfected with miR‐200c mimics or NC mimics and treated with AngII or phosphate‐buffered saline (PBS) for 48 h, and a quantitative analysis of p‐MLC2/MLC2 and cMLCK/GAPDH was conducted; (F‐H) The protein expression levels of cMLCK, MLC2 and p‐MLC2 were detected using Western blotting in NCMs transfected with miR‐200c inhibitor or NC inhibitor and treated with AngII or PBS for 48 h, and a quantitative analysis of p‐MLC2/MLC2 and cMLCK/GAPDH was conducted. (All data are presented as the means ± SD; n = 4 samples per experimental group; *P < 0.05 vs mimics NC or inhibitor NC/PBS; ^#^ *P* < 0.05 vs mimics NC or inhibitor NC/AngII)

**Figure 6:**
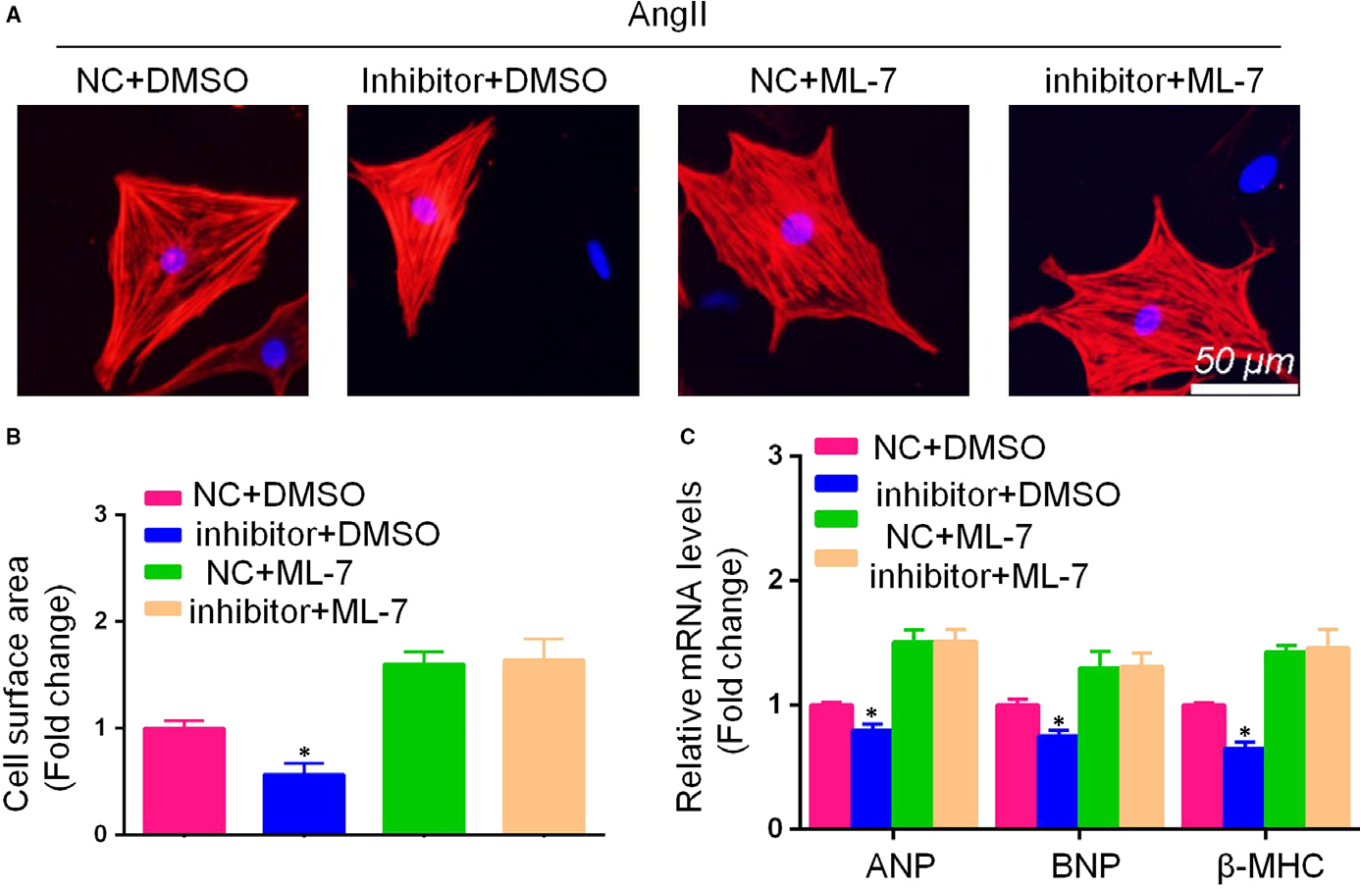
Inhibition of myosin light chain kinase (MLCK) reversed the effect of miR‐200c inhibitor on AngII‐induced cardiomyocyte hypertrophy. (A,B) Representative images and cross‐sectional area of neonatal rat cardiomyocytes in the indicated groups for 48 h (scale bar = 50 μm, n = 100+ cells); C, mRNA expression of the hypertrophic markers atrial natriuretic peptide (ANP) and β‐myosin heavy chain (β‐MHC) in each group. (All data are displayed as the means ± SD; n = 4 samples per experimental group; *P < 0.05 vs inhibitor NC)

To reveal potential miRNAs with the potential to regulate the expression of MLCK, Targetscan was used to predict possible miR‐200c targets based on the reverse complementarity of the rno‐miR‐200c seed sequence is reverse complementary to the seed‐match sequence in the 3′‐UTR of MLCK. To experimentally validate the status of MLCK as a direct target of miR‐200c, we constructed luciferase reporter vectors containing either the wild‐type MLCK 3′‐UTR or version containing several mutations in the miR‐200c binding sites (Figure 5A). The vectors and the miR‐200c mimics were then cotransfected into HEK 293 cells. As shown in Figure 5B, the luciferase activity of the wild‐type vector was significantly reduced, (approximately 50%,) by the miR‐200c mimics, whereas the luciferase activity of the mutant vector was not substantially altered. This result indicated that miR‐200c directly binds to the 3′‐UTR of MLCK (Figure 6).

As mentioned above, miR‐200c directly targets MLCK. To determine whether or not miR‐200c exerts its effects in hypertrophic cardiomyocytes via regulation of the MLCK signalling pathway, we performed Western blotting analyses, which analysis revealed that the expression of MLCK and the phosphorylation of MLC2 were significantly reduced in AngII‐induced NCMs transfected with miR‐200c mimics compared with those transfected with miR‐NC. In contrast, the expression of MLCK and the phosphorylation of MLC2 were significantly increased by miR‐200c inhibitor pretreatment in AngII‐induced NCMs. No obvious change in the expression of MLC2 was detected. These data suggest that miR‐200c negatively regulates MLCK and its downstream mediators, including the MLC2 signalling pathway.

### 3.6 Inhibiting MLCK reversed the effects of the miR‐200c inhibitor on AngII‐induced hypertrophic cardiomyocytes

The above results indicated that miR‐200c promotes CH by inhibiting MLCK signalling cascades. To provide further evidence of this, we cultured NCMs that had been previously transfected with the miR‐200c inhibitor with an MLCK inhibitor, ML‐7, then treated these with AngII for 48 hours. Analysis of the mRNA levels of hypertrophic markers (ANP, BNP and β‐MHC) revealed that ML‐7 treatment reversed the cardioprotective effects of the miR‐200c inhibitor on AngII‐induced hypertrophic cardiomyocytes. Similar effects were observed for the cardiomyocyte surface area. These data suggest that miR‐200c inhibitor‐mediated CH is largely dependent on the activation of MLCK signalling.

## 4 DISCUSSION

In this study, we predicted miR‐200c to be a candidate miRNA to regulate cMLCK during CH. The following observations were made: (a) miR‐200c acted as a new pro‐hypertrophic miRNA; (b) the down‐regulation of miR‐200c inhibited cardiomyocyte apoptosis and reduced the production of ROS; (c) miR‐200c directly targeted MLCK; (d) the suppression of miR‐200c ameliorated CH via activation of the MLCK signalling pathway.

To date, a number of miRNAs have been demonstrated to regulate the expression levels of genes that govern the physiological and pathological processes that underlie CH. miRNAs are often maintained at a stable level under normal physiological conditions. However, changes in the expression levels of miRNAs have often been observed in nearly all cardiovascular disorders.^26^ Several pro‐hypertrophic miRNAs, such as miR‐208, miR‐195, miR‐23a, miR‐106a and miR‐199, have been shown to lead to physiological CH.^27^, ^28^ Certainly, anti‐hypertrophic miRNAs have also been identified, including miR‐22 and miR‐133.^29^ Mir‐200c was first found to be expressed at a low level in non‐small‐cell lung cancer A549 cells, and miR‐200c has been shown to play an important role in epithelial mesenchymal transition.^30^ More recently, researchers have investigated the role of miR‐200c in cardiovascular diseases. The level of miR‐200c was found to be increased in a mouse model of doxorubicin‐induced cardiotoxicity.^31^ The down‐regulation of miR‐200c in cardiomyocytes exerted protective effects against hypoxia via the up‐regulation of GATA‐4, a zinc‐finger transcription factor.^32^ Moreover, the inhibition of miR‐200c attenuated diabetes‐associated CH by modulating the expression of dual‐specific phosphatase‐1 (DUSP‐1), which has been reported to inactivate the activity of mitogen‐activated protein kinases (MAPKs) such as extracellular regulated protein kinases, c‐Jun N‐terminal kinase and p38 in diabetic cardiomyopathy (DCM).^33^ Mitogen‐activated protein kinases are up‐regulated in DCM and CH. miR‐200c has also been shown to play a role in the regulation of glutamine metabolism, which is involved in antioxidative processes during ischaemia/reperfusion injury.^34^ In present, Xi Fang et al showed that down‐regulation of miR‐200a was able to attenuate CH, inhibiting the release of peroxisome proliferator‐activated receptor gamma activated can increases CH.^35^ The above‐mentioned results support the role of miR‐200c in cardioprotection. Here, we have identified the effects of miR‐200c in CH. First, miR‐200c was found to be markedly up‐regulated in both pressure overload‐induced hypertrophic myocardium and in AngII‐induced hypertrophic cardiomyocytes. We also observed that the knockdown of miR‐200c with a specific inhibitor attenuated the degree of cardiomyocyte hypertrophy induced by AngII, whereas the overexpression of miR‐200c almost completely reversed the effects of the miR‐200c inhibitor, which suggests that miR‐200c plays a crucial role in CH.

Apoptosis is a common phenomenon observed in cases of CH. Pathological myocardial hypertrophy is mainly caused by the activation of AngII and other hypertrophic factors, which, stimulate apoptotic genes to promote cardiomyocyte apoptosis and decrease the myocardial contraction force and, lead to an imbalance in the regulation of negative feedback that results from the proliferation and apoptosis of cardiomyocytes.^36^ Chen et  al indicated that the down‐regulation of miR‐200c suppressed hypoxia‐induced cardiomyocyte apoptosis by targeting GATA‐4.^32^ In our study, we found that the down‐regulation of miR‐200c potentially exerted an anti‐apoptotic effect in AngII‐induced hypertrophic cardiomyocytes. In contrast, the up‐regulation of miR‐200c produced the opposite effects.

Reactive oxygen species have been proposed to contribute to the deterioration of cardiac function in the development of heart disease. An imbalance between ROS generation and elimination is a large source of myocardial injury in CH, which is characterized by the excessive intracellular production and accumulation of ROS, specifically superoxide (O^2−^) and hydrogen peroxide, and an increase in enzymatic sources of ROS. As ROS production continues to increase, the expression and activity of antioxidant enzymes may reach saturation and actually decrease, increasing the level of oxidative damage.^37^ Numerous studies have proven that ROS overproduction is present in AngII‐treated CMs. Correspondingly, our current data suggested that the down‐regulation miR‐200c reduced ROS production and lipid peroxidation, increased SOD activity and decreased the MDA level. In endothelial cells, ROS‐induced miR‐200c overexpression promoted cell growth arrest, senescence and apoptosis by targeting zinc‐finger E‐box binding homeobox 1,^38^ suggested that miR‐200c may be involve in ROS‐mediated cardiovascular dysfunction. Moreover, miR‐200c can alleviate myocardial ischaemia/reperfusion mainly by inhibiting ROS production, as miR‐200c regulates target gene glutaminase, which is an essential antioxidant.^34^ Here, we demonstrated for the first time that a miR‐200c inhibitor prevented ROS production in AngII‐induced hypertrophic cardiomyocytes.

The possible mechanisms by which miR‐200c regulates CH may be associated with its downstream targets in the MLCK signalling pathway. Previous studies have revealed that cMLCK is involved in the regulation of ventricular myosin light chain 2 (MLC‐2v) phosphorylation, sarcomere organization and cardiomyocyte contraction.^39^ The phosphorylation of MLC‐2v has been shown to play an important regulatory role in maintaining normal cardiac function, with increasing in MLC‐2v phosphorylation likely inhibiting CH and HF by contributing to enhanced contractile performance and efficiency.^15^, ^39^ In an earlier study, we found that MLCK and p‐MLC2 were involved in CH due to their degradation by AngII.^40^ The overexpression of cMLCK in cardiac myocytes promotes sarcomere organization, which is an adaptive response to hypertrophic stimuli during early CH, while the knockdown of cMLCK resulted in sarcomeric disorganization.^41^ Moreover, the expression level of cMLCK is reduced in animal models of MI.^10^ After 1 week, a reduction in the levels of cMLCK and phosphorylated MLC2v were also demonstrated in the hearts subject to pressure overload induced by thoracic aortic constriction (TAC) surgery.^11^ In addition, the knockdown of cMLCK in *Mylk3*‐KO mice was associated with HF, which suggested that cMLCK plays a pivotal role in the transition from compensated to decompensated hypertrophy via sarcomeric disorganization.^42^ The present findings suggest a protective role for cMLCK against cardiac stress. Consistent with the above study, we found that the expression of MLCK was reduced during CH both in vivo and in vitro. In this study, we have shown that the expression of MLCK is negatively regulated by miR‐200c, which may be one of the mechanisms by which miR‐200c promotes cardiomyocyte hypertrophy.

In summary, our study demonstrated that the levels of miR‐200c are increased during CH. The down‐regulation of miR‐200c provides considerable protective effects against AngII‐induced CH in cardiomyocytes via the targeting of MLCK and by decreasing apoptosis and ROS production. Our study suggests that miR‐200c may serve as a potential therapeutic target for the treatment of CH.

## CONFLICT OF INTEREST

All authors declare no conflicts of interest.
