# Correction to Down‐regulation of miR‐200c attenuates AngII‐induced cardiac hypertrophy via targeting the MLCK‐mediated pathway

**DOI:** 10.1111/jcmm.17724

**Published:** 2023-07-03

In Hu et al.,^1^ the published article contains errors in Figure 4E,F. The figure of group inhibitor + PBS in Figure 4E was mis‐uploaded. The corrected Figure 4 is below. The authors confirmed that all results and conclusions of this article remain unchanged.

**FIGURE 4:**
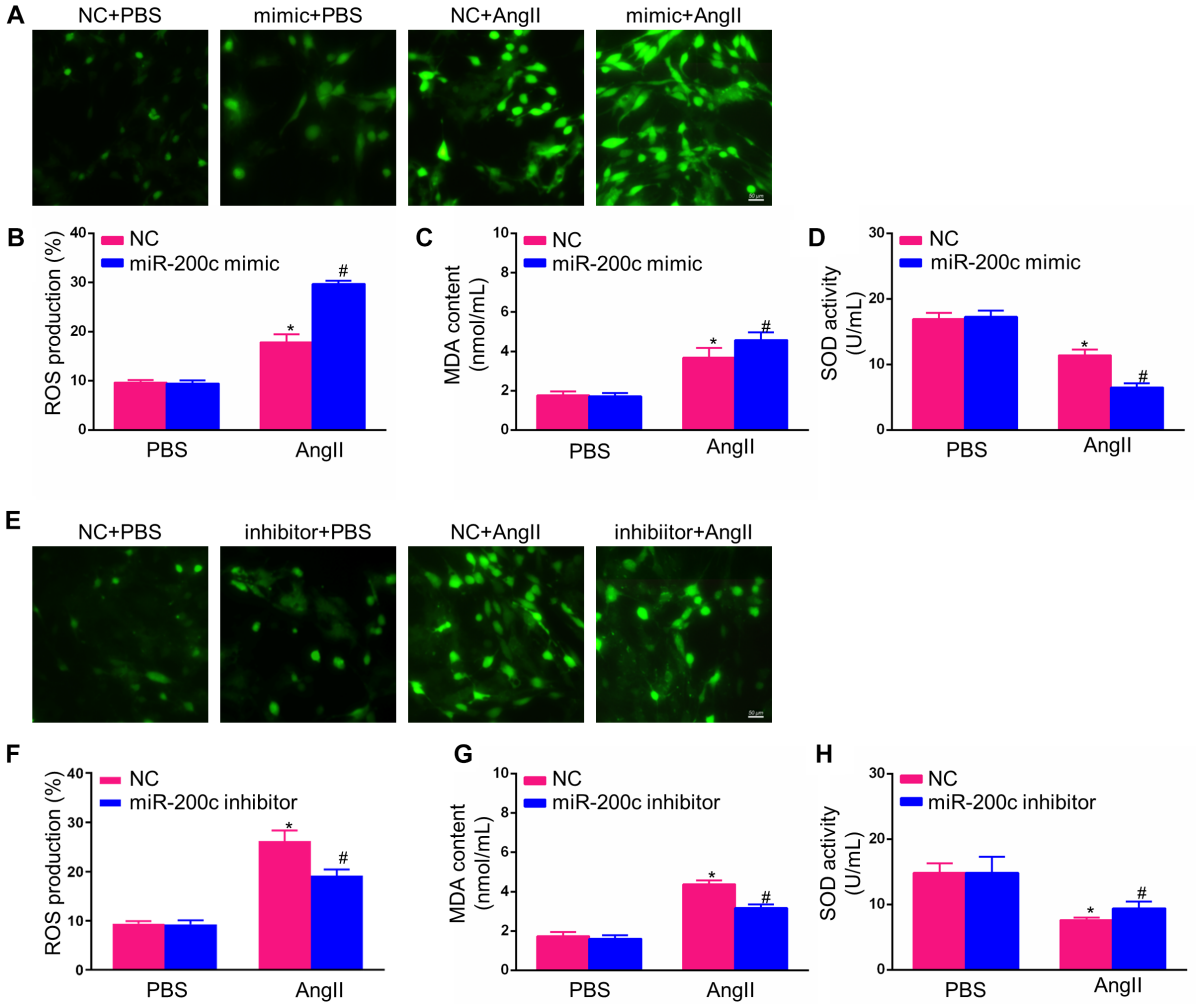
MiR‐200c‐mediated cardiac hypertrophy was associated with reactive oxygen species (ROS) regulation. (A, B) Representative photomicrographs showing ROS accumulation, as determined using the 2,7‐dichlorodihydrofluorescein diacetate (DCFH‐DA) probe in neonatal rat cardiomyocytes (NCMs) transfected with a miR‐200c mimics or NC mimics and treated with AngII or phosphate‐buffered saline (PBS) for 48 h (scale bar = 50 μm); (C, D) Relative levels of malondialdehyde (MDA) content and superoxide dismutase (SOD) activity in NCMs transfected with miR‐200c mimics or NC mimics and treated with AngII or PBS for 48 h; (E, F) Representative photomicrographs showing ROS accumulation, as determined by the DCFH‐DA probe under a fluorescence microscope in NCMs transfected with miR‐200c inhibitor or NC inhibitor and treated with AngII or PBS for 48 h (scale bar = 50 μm); (G, H) Relative levels of MDA content and SOD activity in NCMs transfected with miR‐200c inhibitor or NC inhibitor and treated with AngII or PBS for 48 h. (All data are displayed as the means ± SD; *n* = 4 samples per experimental group; *p < 0.05 vs. NC mimics or NC inhibitor/PBS; #*p* < 0.05 vs. NC mimics or NC inhibitor/AngII).
